# DNA-Methylation Patterns in Trisomy 21 Using Cells from Monozygotic Twins

**DOI:** 10.1371/journal.pone.0135555

**Published:** 2015-08-28

**Authors:** M. Reza Sailani, Federico A. Santoni, Audrey Letourneau, Christelle Borel, Periklis Makrythanasis, Youssef Hibaoui, Konstantin Popadin, Ximena Bonilla, Michel Guipponi, Corinne Gehrig, Anne Vannier, Frederique Carre-Pigeon, Anis Feki, Dean Nizetic, Stylianos E. Antonarakis

**Affiliations:** 1 Department of Genetic Medicine and Development, University of Geneva, Geneva, Switzerland; 2 National Center of Competence in Research *Frontiers in Genetics* Program, University of Geneva, Geneva, Switzerland; 3 Stem Cell Research Laboratory, Department of Obstetrics and Gynecology, Geneva University Hospitals, Geneva, Switzerland; 4 Department of Obstetrics and Gynecology, Hôpital Cantonal Fribourgeois, Fribourg, Switzerland; 5 Centre Hospitalier Universitaire Reims, Service de Genetique et de Biologie de la Reproduction, CECOS, Hopital Maison Blanche, F-51092 Reims, France; 6 The Blizard Institute, Barts and The London School of Medicine, Queen Mary University of London, 4 Newark Street, London E1 2AT, United Kingdom; 7 Lee Kong Chian School of Medicine, Nanyang Technological University, Unit 04–11, Proteos Building, 61 Biopolis Drive, Singapore 138673, Singapore; 8 iGE3 institute of Genetics and Genomics of Geneva, University of Geneva, Geneva, Switzerland; University of Bonn, Institut of experimental hematology and transfusion medicine, GERMANY

## Abstract

DNA methylation is essential in mammalian development. We have hypothesized that methylation differences induced by trisomy 21 (T21) contribute to the phenotypic characteristics and heterogeneity in Down syndrome (DS). In order to determine the methylation differences in T21 without interference of the interindividual genomic variation, we have used fetal skin fibroblasts from monozygotic (MZ) twins discordant for T21. We also used skin fibroblasts from MZ twins concordant for T21, normal MZ twins without T21, and unrelated normal and T21 individuals. Reduced Representation Bisulfite Sequencing (RRBS) revealed 35 differentially methylated promoter regions (DMRs) (Absolute methylation differences = 25%, FDR < 0.001) in MZ twins discordant for T21 that have also been observed in comparison between unrelated normal and T21 individuals. The identified DMRs are enriched for genes involved in embryonic organ morphogenesis (FDR = 1.60 e -03) and include genes of the *HOXB* and *HOXD* clusters. These DMRs are maintained in iPS cells generated from this twin pair and are correlated with the gene expression changes. We have also observed an increase in DNA methylation level in the T21 methylome compared to the normal euploid methylome. This observation is concordant with the up regulation of DNA methyltransferase enzymes (DNMT3B and DNMT3L) and down regulation of DNA demethylation enzymes (TET2 and TET3) observed in the iPSC of the T21 versus normal twin. Altogether, the results of this study highlight the epigenetic effects of the extra chromosome 21 in T21 on loci outside of this chromosome that are relevant to DS associated phenotypes.

## Introduction

Genomic aneuploidy is a common cause of human genetic disorders that often results in dysregulation of gene expression patterns. A classic example of genomic aneuploidy is trisomy 21 (T21), which results to a collection of phenotypes known as Down syndrome (DS)[1]. Using monozygotic (MZ) twins discordant for T21, we have recently shown that the differential expression in T21 is organized in gene expression dysregulation domains (GEDDs) across the genome [2]. The DS associated phenotypes can be the result of the extra chromosomal material *per se*, or specific genes on chromosome 21. Some of the DS phenotypes (e.g., cognitive impairment) are consistently present in all DS individuals, while others show incomplete penetrance [1]. For the variable phenotypes with incomplete penetrance allele specific trisomy may be involved. In accordance with this hypothesis, we have reported the association of chromosome 21 SNPs and CNVs with an increased risk of congenital heart defect (CHD) in DS [3]. Alternatively, the variability of the phenotypes or the severity of the syndrome may be due to changes of the epigenetic landscape. Indeed, epigenetic alterations are known to contribute to human health and diseases [4–8]. Epigenetic modifications may also explain why MZ twins are not always phenotypically identical [9]. DNA methylation is among the best-studied epigenetic modifications thus far [8]. Potentially, DNA methylation could be involved in the response to gene dosage imbalances, for example in the case of aneuploidies. However, it is unclear if specific epigenetic events are needed for the development of DS phenotypes. There have been a few studies investigating the DNA methylation states in DS. In 2009, Zhang *et al* investigated chromosome 21 genes in DS and non-DS cells, and have found differences in promoter methylation of chromosome 21 genes [10]. Moreover, Kerkel *et al*., investigated DNA methylation profiling of 27,000 CpGs genome-wide using methylation arrays in peripheral blood leukocytes and T lymphocytes and reported gene specific abnormalities of CpG methylation in DS cases [11]. Although the number of CpGs investigated in this study (27,000 CpGs) is only a small fraction of CpGs, the results highlighted the importance of studying DNA methylation in aneuploid cases on a larger scale. Furthermore, Jin et al., investigated DNA methylation alterations associated with DS, in placenta villi of 11 DS cases and 6 normal controls [12], and identified genes with differential DNA methylation. However, it is not clear if these observations are specific to T21, or the results of the interindividual variability [13].

In the present study we hypothesized that there are epigenetic modifications in T21 that could potentially be linked to DS associated phenotypes. We applied Reduced Representation Bisulphite Sequencing (RRBS) to profile DNA methylation changes in a pair of rare MZ twins discordant for T21 [2,14] and two pairs of rare MZ twins concordant for trisomy 21, but discordant for CHD. We also studied two pairs of unaffected MZ twins without T21, as well as unrelated normal and T21 cases as controls. In addition, we have used iPS cells generated from MZ twins discordant for T21 to determine whether changes in DNA methylation in T21 are maintained during reprogramming. The results of this study identified epigenetic changes in genes involved in embryonic organ morphogenesis in MZ twins discordant for T21, and genes with potential involvement in heart development in T21 MZ twins discordant for CHD.

## Material and Methods

### Description of samples

Primary fetal fibroblasts from the MZ twins discordant for T21 were derived from skin tissue collected post mortem (Table 1, samples 1, 2, 3 and 4) [2,14]. Primary skin fibroblasts were also obtained from a pair of MZ twins concordant for T21, but discordant for CHD (VSD, ventricular septal defect in particular) (Table 1, samples 5 and 6). Informed consent was obtained for all these twin samples, and the Geneva University Ethics Committee approved the study. Additionally, we used amniocytes from a pair of MZ twins concordant for T21, but discordant for CHD (AVSD, Atrio Ventricular Septal Defect) (Table 1, samples 7 and 8). This twin set was obtained from Institut mère-enfant Alix de Champagne in France [15]. Moreover, primary fibroblasts from two pairs of healthy unaffected MZ twins (both normal euploid twins) have been used (Table 1, samples 9, 10, 11, and 12). Samples 9 and 10 have been obtained from the Geneva GenCord project [13], and samples 11 and 12 have been obtained from the TwinsUK resource (http://www.twinsuk.ac.uk) at the King's College London. The primary fibroblasts from unrelated T21 and normal individuals were taken from Prandini *et al* [16]. In addition to these samples, we also studied induced pluripotent stem cells (iPS) generated from the fetal skin fibroblasts of the MZ twins discordant for T21 [17] (Table 1, samples 15, 16 and 17). Table 1 shows the description of the samples used in this study.

**Table 1:** Description of samples used for DNA methylation analyses.

Sample set	Sample ID	Gender	Description	Age*	Passage
MZ twins discordant for T21 (Replicate 1)	Sample 1	Female	Normal primary fetal skin fibroblasts	13 WG	P13
MZ twins discordant for T21 (Replicate 1)	Sample 2	Female	T21 primary fetal skin fibroblasts	13 WG	P13
MZ twins discordant for T21 (Replicate 2)	Sample 3	Female	Normal primary fetal skin fibroblasts	13 WG	P13
MZ twins discordant for T21 (Replicate 2)	Sample 4	Female	T21 primary fetal skin fibroblasts	13 WG	P13
T21 MZ twins discordant for VSD	Sample 5	Male	T21 with VSD primary skin fibroblasts	4 years	P5
T21 MZ twins discordant for VSD	Sample 6	Male	T21 without VSD primary skin fibroblasts	4 years	P5
T21 MZ twins discordant for AVSD	Sample 7	Male	T21 with AVSD amniocytes	17 WG	NA
T21 MZ twins discordant for AVSD	Sample 8	Male	T21 without AVSD amniocytes	17 WG	NA
Normal MZ twins pair 1	Sample 9	Female	Normal primary fetal skin fibroblasts	Fetal	P5
Normal MZ twins pair 1	Sample 10	Male	Normal primary fetal skin fibroblasts	Fetal	P5
Normal MZ twins pair 2	Sample 11	Female	Normal primary skin fibroblasts	53 years	P8
Normal MZ twins pair 2	Sample 12	Female	Normal primary skin fibroblasts	53 years	P9
Unrelated normal and T21	Sample 13	Female	T21 primary skin fibroblasts	3 days	P14
Unrelated normal and T21	Sample 14	Female	Normal primary skin fibroblasts	2 days	P14
MZ twins discordant for T21 iPS cells	Sample 15	Female	Normal iPS cells	13 WG	P10
MZ twins discordant for T21 iPS cells	Sample 16	Female	T21 iPS cells	13 WG	P10
MZ twins discordant for T21 iPS cells	Sample 17	Female	T21 iPS cells	13 WG	P10

*WG, Weeks of Gestation.

NA, Not Applicable

### Cell culture

All the primary fibroblast cells were grown in DMEM GlutaMAX media (Life Technologies) enriched with 10% fetal bovine serum (FBS) (Life Technologies) and 1% penicillin/streptomycin/fungizone mix (Amimed, BioConcept) at 37°C in a 5% CO_2_ incubator. All the iPS cells were grown as previously described [17].

DNA extraction was performed using Qiagen DNeasy Blood and Tissue Kit from the primary skin fibroblast cells. Total RNA was extracted using the TRIzol reagent (Life Technologies) according to the manufacturer’s instructions. RNA quality was checked on the Agilent 2100 Bioanalyzer and quantity was measured on a Qubit instrument (Life Technologies).

### RRBS library preparation and sequencing

We prepared RRBS libraries according to Gu H et al., with some modifications [18]. Briefly, 2 μg of genomic DNA was digested overnight at 37°C with 2 μl 20U/μl MspI and 5μl NEB4 (New England Biolabs Inc.) in a total reaction volume of 50 μl, followed by heat inactivation at 80°C for 20 minutes. Subsequently the end repair and A tailing were performed by adding 2 μl dNTP mix (10mM), 2 μl 5U/ μl Klenow fragment (New England Biolabs Inc.), and 4μl of NEB2 (New England Biolabs Inc.) in a total reaction volume of 40 μl. This reaction was incubated at 30°C for 20 minutes, followed by 20°C for 37 minutes. Heat inactivation was performed at 75°C for 20 minutes. The end-repaired and A tailed molecules were ligated to the methylated version of the Illumina adapters; ilAdap Methyl PE1: ACACTCTTTCCCTACACGACGCTCTTCCGATC*T (all C’s are methylated, * = phosphorothioate bond) and ilAdap Methyl PE2: GATCGGAAGAGCGGTTCAGCAGGAATGCCGA*G (all C’s are methylated, 5’ phosphate, * = phosphorothioate bond). The ligation was done using 1μl T4 DNA ligase (2,000 U/μl) (New England Biolabs Inc.), 2μl T4 ligase buffer (10X) (New England Biolabs Inc.), and 1μl methylated adapters (paired end adapters)(15 μM) in a total reaction volume of 20μl. The reaction was incubated in a thermocycler at 16°C overnight. The adapter ligated DNA was then purified by phenol extraction and ethanol precipitation and dissolved in 15 μl EB buffer. Size selection of the adapter ligated DNA fragments (170 bp to 350 bp) was done by electrophoresing the 15 μl ligation reaction in a 2.5% NuSieve GTG agarose gel (Lonza). We subsequently purified the DNA using a Qiagen Qiaquick Gel Extraction kit as described in the manufacturer’s instructions. We then used 20 μl of this purified DNA in the sodium bisulfite conversion step, which was performed using the QIAGEN Epitect Bisulfite Kit (Qiagen, Valencia CA, USA). The purified bisulfite treated DNA was then PCR amplified in a reaction containing 100 μl KAPA2G Robust DNA polymerase mixture (KAPA biosystems), 40μl bisulphite treated DNA, 0.5 mM ilPCR PE1 primer (AAT GAT ACG GCG ACC ACC GAG ATC TAC ACT CTT TCC CTA CAC GAC GCT CTT CCG ATC* T; * = phosphorothioate bond), 0.5 mM ilPCR PE2 primer (CAA GCA GAA GAC GGC ATA CGA GAT CGG TCT CGG CAT TCC TGC TGA ACC GCT CTT CCG ATC* T; * = phosphorothioate bond) in a total reaction volume of 200 μl. The PCR mixture were divided amongst eight PCR tubes, and were incubated at 95°C for 2 minutes, followed by 20 cycles of (95°C for 20 seconds and 65°C for 30 seconds and 72°C for 30 seconds), and finally 72°C for 7 minutes. The PCR products were pooled, and purified by adding 360 μl AMPure magnetic beads (Agencourt Bioscience, Beverly, MA). We quantified the purified product using the Qubit fluorometer (Invitrogen). We also checked the template size distribution of the library on Agilent Bioanalyzer (Agilent 2100 Bioanalyzer). We then diluted each library to 10 nM and proceeded to sequence each library (pair ended 100bp) in a single lane on the Illumina HiSeq 2000 according the manufacturer's instructions.

### DNA methylation data processing

The quality of the reads were measured by FASTQC (version 0.10.1) program as implemented in the *trime galore* wrapper (http://www.bioinformatics.babraham.ac.uk). Low quality base calls were removed from the 3' end of the reads before trimming the adapter sequences. Our cut off for base quality control was Phred quality score of 20. Subsequently, *Cutadapt* detected and trimmed the adapter sequences from the 3’ end of reads. Bismark aligner [19] was used for mapping reads against the human genome hg19. For post data analyses purposes, we used an *R* library package methylKit [20] and custom scripts implemented in *R* and Python. We used the MethylKit package to calculate methylation percentages per each single CpG. Percent methylation values for CpG dinucleotides were calculated by dividing the number of methylated Cs by the total coverage on that base. CpGs with at least 20X read coverage and at least a Phred score threshold of 20 were retained for calling CpG methylation. We calculated DNA methylation state at single CpG sites and over genomic features such as promoters, CpG islands, and gene bodies (Exons and Introns).

### DNA methylation level at single CpG sites and genomic regions

DNA methylation level was calculated for each sample both at single CpG sites and genomic regions. Genomic regions (GRCh37/hg19) include CpG islands, CpG shores (defined as 2000bp flanking regions on each side of the CpG island), promoters (defined as -2000bp, +1000bp around the TSS), and gene body (introns and exons), lamina associated domains (LADs), and regions between LAD domains (iLADs) [21]. For scoring DNA methylation in our analyses, each individual CpG was required to pass a minimum Phred base quality score of 20 and be covered at least by 20 reads. To score DNA methylation over a region, the region was required to contain at least three CpGs each covered by at least 20 reads.

### RNA library preparation, sequencing and data processing

mRNA-seq data were prepared from 5μg of total RNA using the Illumina mRNA-Seq Sample Preparation kit (#RS-100-0801), according to the manufacturer’s instructions. One library per lane was sequenced on an Illumina HiSeq 2000 instrument (paired-end 100bp). Reads were uniquely mapped against the human genome (hg19) using the default parameters of the GEM aligner [22]. Quantile normalization was applied on the RPKM data (Reads Per Kilobase per Million). mRNA-Seq data for fibroblasts of MZ twins discordant for T21 (Samples 1, 2, 3 and 4) were taken from [2]. mRNA-seq data from iPS cells generated from MZ twins discordant for T21 fibroblasts (Samples 15, 16 and 17) were taken from [17].

## Results

In this study we have used MZ twins to evaluate the role of the extra copy of chromosome 21 in epigenetic modifications related to T21. We applied RRBS to quantify DNA methylation at single CpG sites, and over genomic features.

The RRBS method used in this study routinely achieved a bisulfite conversion rate greater than 99.8% for all of the samples studied, as calculated by methylKit [20]. A summary of mapped reads using the BisMark aligner against the human reference genome (hg19) is presented in S1 Table. Two technical replicates for the MZ twins discordant for T21 (replicate 1 and replicate 2) from the same culture, but with independent bisulfite conversion and library preparation were produced. The overall DNA methylation correlation between the two technical replicates was 0.90 for the normal twin (Samples 1 and 3 of Table 1) and 0.91 for T21 twin (Samples 2 and 4 of Table 1) (S1 Fig).

**Fig 1:**
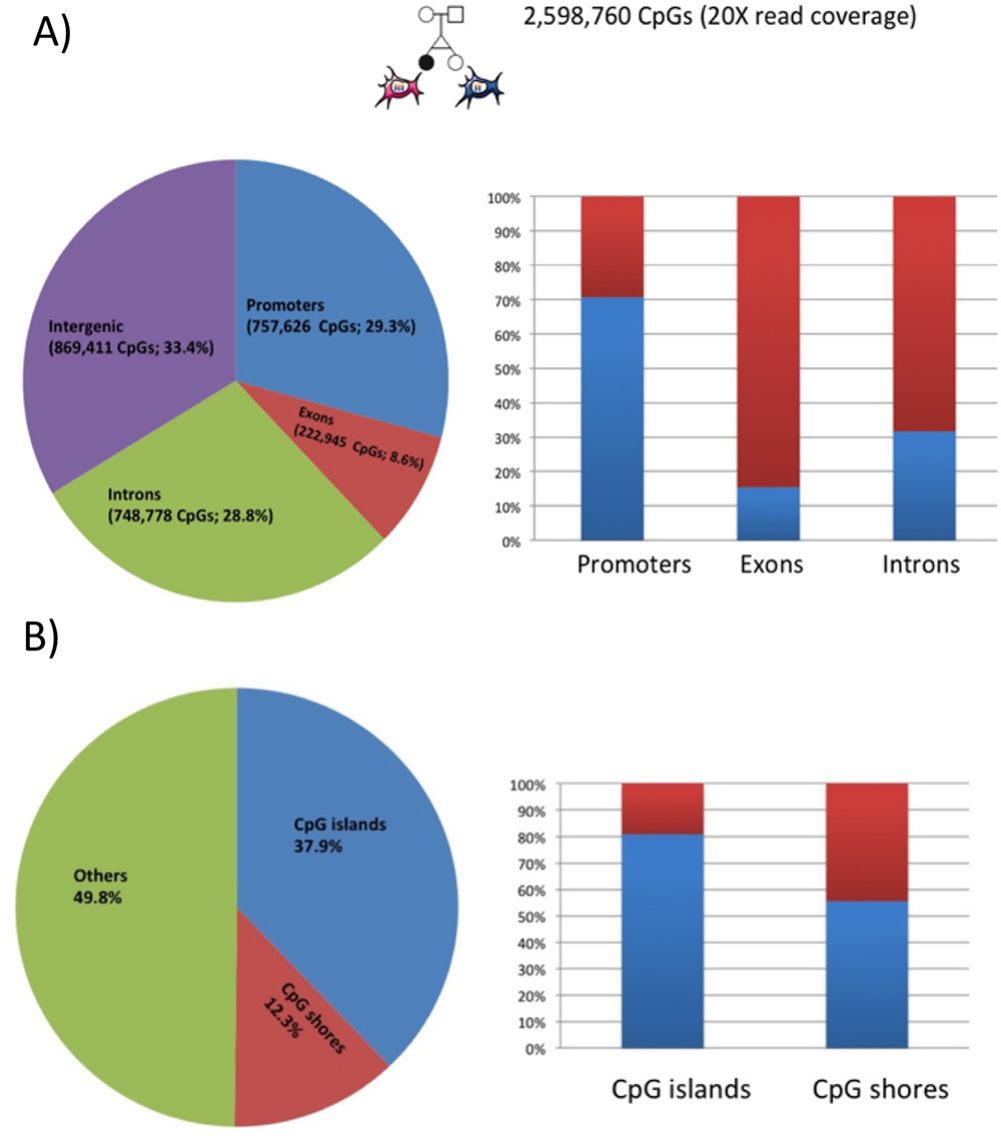
CpGs Coverage of MZ-twins discordant for T21. A) Distributions of covered CpGs (≥20X read coverage) by RRBS in promoters, exons, introns, and intergenic regions are shown in the pie chart. The blue bar in the histogram panel shows how many percentages of the genomic features are covered by RRBS. B) Distributions of covered CpGs (≥20X read coverage) by RRBS in CpG islands.

**Fig 2:**
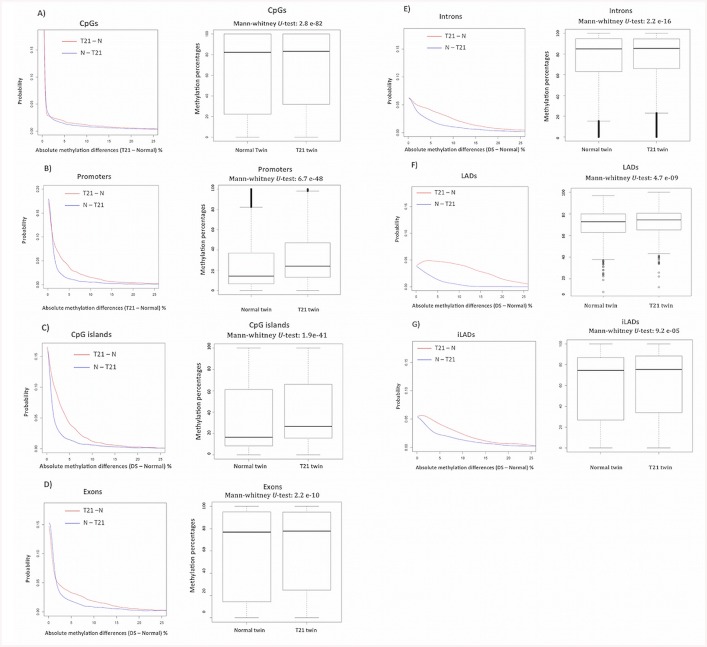
The overall DNA methylation level at single CpG resolution, and over genomic features. The probability density distribution and the box-plot of DNA methylation shows differences between the T21 twin and the normal twin at the level of A) single CpG sites, B) promoters, C) CpG islands, D) Exons, E) Introns, F) LADs, and G) iLADs. The red line in the probability plot shows the probability distribution of T21 twin methylome subtracted from the normal twin methylome (T21—N). While, the blue line shows the probability distribution of the normal twin methylome subtracted from T21 twin methylome (N—T21). DNA hyper-methylation in T21 twin (T21 > Normal) is observed at the level of CpGs, and all the genomic features, but the effect is more pronounced in promoters and CpG islands.

Fig 1 shows the RRBS coverage of CpG sites in the MZ twins discordant for T21. A total of 2,598,760 CpGs (Phred base quality ≥ 20 and base read coverage ≥ 20) have been covered. That includes 70.7% of gene promoters, and 80.8% of CpG islands (Fig 1). Using the MZ twins discordant for T21 (samples 1, 2, 3 and 4 of Table 1) we observed a higher level of DNA methylation in the T21 twin compared to the normal twin. This has been observed both at the level of single CpG sites and the genomic features (Fig 2). Fig 2 shows the probability density distribution for methylation differences between the T21 and normal twin (Fig 2). The DNA methylation levels of promoters, introns, exons, CpG islands, CpG shores, LADs, and iLADs were determined, and the overall methylation levels were compared between MZ twins discordant for trisomy 21 using Mann–Whitney *U* test (Fig 2). There is a significant overall hyper-methylation for all the genomic features analyzed in T21 twin. However, the hyper-methylation of T21 twin compared to the normal twin is even more pronounced in promoters (p = 6.7e-48) and CpG islands (p = 1.9e-41) compared to other genomic regions (Fig 2).

**Fig 3:**
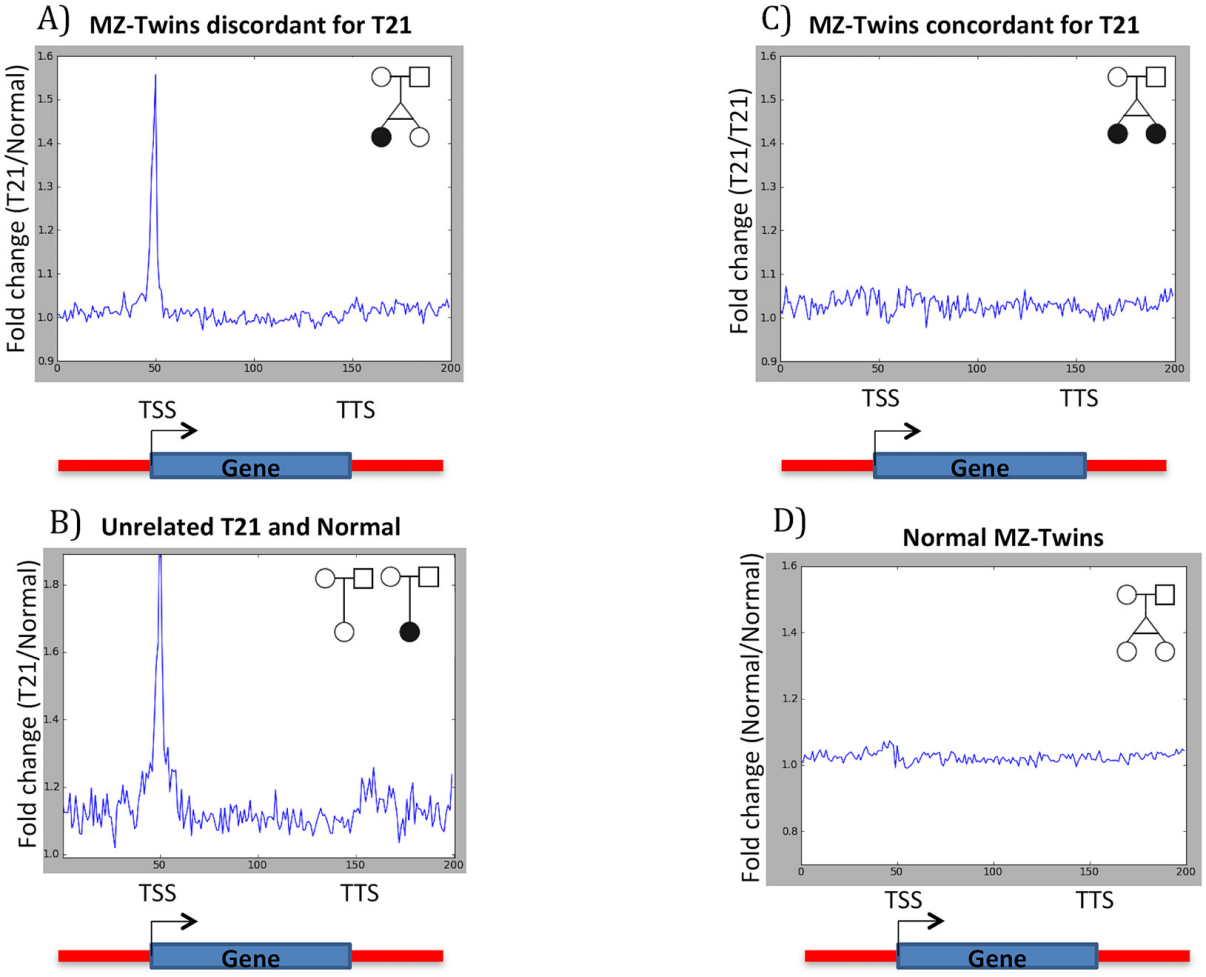
DNA methylation profile over a “Gene Model”. DNA methylation fold change in A) Monozygotic twins discordant for T21, B) Unrelated T21 and normal cases, C) Normal MZ twins, D) T21 MZ twins. TSS and TTS represent transcription start site and transcription termination sites, respectively. The plot shows an increase in the level of DNA methylation around TSS in T21 twin compared to the normal twin.

We next mapped DNA methylation profile to a gene model as described by Laurent *et al* 2010 [23]. This gene model includes all annotated genomic features in the vicinity of all transcribed genes in these twins. This gene model was used to compare the DNA methylation fold changes over the transcription start sites (TTS), gene bodies, transcription termination sites (TTS), and intergenic regions (Fig 3). For this analysis, promoters were defined as -10 kb to +1 kb of the TSS, TTS regions as -1 kb to +10 kb of the TTS, gene body regions as +1 kb from the TSS to -1 kb from the TTS, and intergenic regions consisted of regions not included in the three above mentioned categories [23]. The density of DNA methylation in each region was calculated as the percentage of methyl-cytosine over the total number of covered Cs in that region. We then calculated the fold change of DNA methylation in the T21 twin compared to the normal twin, and observed a higher DNA methylation level (≥ 1.5 fold change) in the gene promoters of T21 twin versus the normal twin (Fig 3). Consistently, the results of unrelated T21 and normal (samples 13 and 14 of Table 1) also showed hyper-methylation around the TSS in T21 compared to the unrelated normal individuals. In contrast normal MZ twins (samples 9 and 10 of Table 1) and MZ twins concordant for T21 (samples 5 and 6 of Table 1) did not show such difference (Fig 3C and 3D).

### Differentially methylated regions (DMRs) in gene promoters

**Fig 4:**
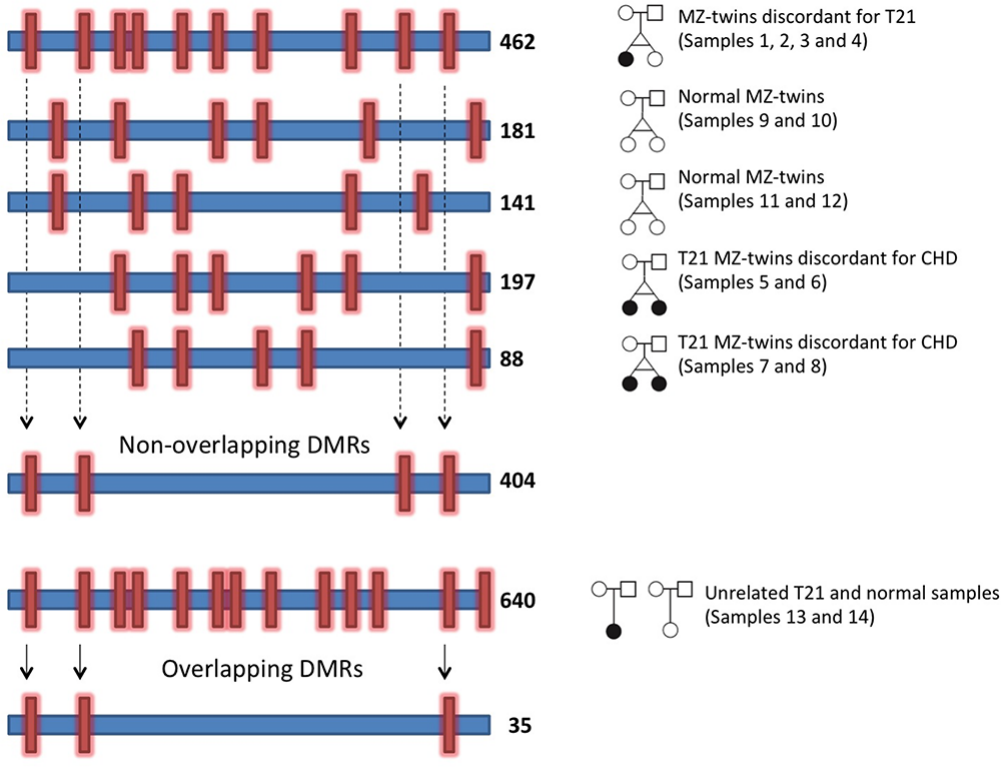
Strategy used to define DMRs of gene promoters induced by the extra copy of chr21 in MZ twins discordant for T21. DMRs are identified in each pair of twins, as well as unrelated normal and T21 cases. DMRs that are common among MZ twins discordant for T21, T21 MZ twins, and normal MZ twins have been excluded from the final candidate list. Subsequently, from this reduced list of DMRs, those that have been observed in unrelated T21 and normal individuals were selected as T21 induced candidate DMRs for further analyses. Each horizontal blue bar represents one pair of sample. The red vertical bars show DMRs. The numbers on the right side of each blue bar shows the number of identified DMRs in each pair of sample.

Since the RRBS method primarily enriches for promoter regions [24], we then focused in identifying differentially methylated regions (DMRs) in gene promoters in the MZ twins discordant for T21. Each promoter region was scored for being DMR using the Fisher's exact test. The *P* values were adjusted for multiple testing using the sliding linear model (SLIM) method that corrects *P* values to q-values as implemented in the methylKit package [20]. Promoters that met or exceeded a q-value of 0.001 and at least 25% absolute methylation differences were reported as being differentially methylated. Fig 4 illustrates the strategy that we have used in order to identify differentially methylated gene promoters in T21. DMRs in gene promoters from hg19 RefSeq genes (http://genome.ucsc.edu) were identified in each pair of twins and unrelated T21 and normal individuals. We have initially identified 462 gene promoters in MZ twins discordant for T21 that are differentially methylated (sample 1 and sample 3 versus sample 2 and sample 4). S2 Table represents the number of common DMRs observed in each sample set pair-wise comparisons (S2 Table). We have excluded any of the 462 DMRs identified in MZ twins discordant for T21 that have been also identified in at least one of the normal twins or T21 concordant twins. This was done in order to only keep those DMRs that are most likely to be due to the extra copy of chromosome 21. This filtering step resulted in 404 DMRs out of 462 to be uniquely present in MZ twins discordant for T21 (Fig 4). The majority of these DMRs are hypermethylated in T21 twins compared to the normal twin (353 gene promoters are hypermethylated and 51 gene promoters are hypomethylated).

Since DMRs identified in MZ twins discordant for T21 are likely to be DMRs in unrelated T21 and normal individuals, we only included those DMRs that were also differentially methylated in unrelated T21 and normal cases, as well (Fig 4). This filtering step identified 35 common DMRs that were kept for further analyses. These DMRs mapped to 37 human Refseq genes (hg19, Table 2). We next performed gene ontology (GO) analyses [25,26] on the 37 genes for which a DMR was identified in their promoter regions. Table 3 shows the significant GO terms and the associated genes in each category. Interestingly, the significant GO terms are linked to embryonic organ morphogenesis and development (Table 3). All the genes in this category are hyper-methylated in the T21 compared to the normal twin. We observed that the majority of the genes in this category, such as *HOXD3*, *HOXD10*, *HOXD12*, *HOXB5*, and *HOXB6* were also differentially methylated in iPS cells generated from primary fetal skin fibroblasts (samples 15, 16 and 17). Table 4 shows the absolute methylation differences, and the log_2_ ratio of fold change in gene expression for the significant GO term in MZ twins discordant for T21 fibroblast cells and MZ twins discordant for T21 iPS cells (Table 4). Moreover, a comparison of these results for genes involved in embryonic organ morphogenesis with previous studies of DNA methylation in DS is shown in S3 Table. Interestingly, the pattern of differentially methylated region is also observed for *HOXD10*, *HOXB5*, *HOXB6 and HOXD12* across multiple studies in different tissues [11,12,27–29] (S3 Table). Additionally, we analyzed the enrichment of histone marks and transcription factor binding sites (TFBS) in 37 gene promoters for which a DMR was identified in MZ twins discordant for T21. Table 5 shows the significant enrichment signals for histone marks (ENCODE_Histone_Modifications_2015) and TFBS (ENCODE_TF_ChIP-seq_2015) [30] in ENCODE human cell lines based on the program *Enrichr* [31]. As shown in Table 5, this analysis confirms that the reported DMRs are targets of a common set of transcription factors and epigenetic regulators, including CTCF and EZH2 binding sites, and H3K27me3, H3K4me1 and H2AFZ chromatin marks.

**Table 2:** DMRs in MZ twin discordant for T21.

Chr.	Start	End	Meth (T21-N)*	Gene name	Gene description
chr1	24016268	24019268	68.33	*RPL11*	Ribosomal protein L11
chr1	115123265	115126265	33.29	*BCAS2*	Breast carcinoma amplified sequence 2
chr1	115123265	115126265	33.29	*DENND2C*	DENN/MADD domain containing 2C
chr1	155909479	155912479	26.17	*RXFP4*	Relaxin/insulin-like family peptide receptor 4
chr2	26780566	26783566	26.11	*OTOF*	Otoferlin
chr2	176962529	176965529	55.30	*HOXD12*	Homeobox D12
chr2	176979491	176982491	62.16	*HOXD10*	Homeobox D10
chr2	177026804	177029804	35.19	*HOXD3*	Homeobox D3
chr3	183722125	183725125	-33.80	*ABCC5*	ATP-binding cassette, sub-family C
chr3	195941382	195944382	25.26	*SLC51A*	Solute carrier family 51
chr5	79550898	79553898	49.96	*SERINC5*	Serine incorporator 5
chr5	140798536	140801536	25.12	*PCDHGA1*	Protocadherin gamma subfamily A
chr5	179498118	179501118	-26.77	*RNF130*	Ring finger protein 130
chr7	129140319	129143319	43.34	*SMKR1*	Small lysine-rich protein 1
chr7	148934741	148937741	47.16	*ZNF212*	Zinc finger protein 212
chr8	104381742	104384742	31.30	*CTHRC1*	Collagen triple helix repeat containing 1
chr9	35904188	35907188	-25.15	*HRCT1*	Histidine rich carboxyl terminus 1
chr9	131682561	131685561	-39.51	*PHYHD1*	Phytanoyl-coa dioxygenase domain 1
chr9	132403448	132406448	40.11	*ASB6*	Ankyrin repeat and SOCS box containing 6
chr9	133882503	133885503	30.65	*LAMC3*	Laminin, gamma 3
chr10	100027007	100030007	41.49	*LOXL4*	Lysyl oxidase-like 4
chr11	17564963	17567963	41.50	*USH1C*	Usher syndrome 1C
chr15	74464086	74467086	38.36	*ISLR*	Immunoglobulin superfamily leucine-rich
chr15	91471409	91474409	39.87	*HDDC3*	HD domain containing 3
chr16	68266402	68269402	33.39	*ESRP2*	Epithelial splicing regulatory protein 2
chr16	73124247	73127247	31.28	*HCCAT5*	Hepatocellular carcinoma transcript 5
chr16	75527926	75530926	52.13	*CHST6*	Carbohydrate
chr17	8868029	8871029	25.36	*PIK3R5*	Phosphoinositide-3-kinase
chr17	46671319	46674319	45.27	*HOXB5*	Homeobox B5
chr17	46671319	46674319	45.27	*HOXB6*	Homeobox B6
chr19	4866780	4869780	44.22	*PLIN3*	Perilipin 3
chr19	36247043	36250043	27.12	*HSPB6*	Heat shock protein B6
chr19	46848250	46851250	25.66	*PPP5C*	Protein phosphatase 5, catalytic subunit
chr19	55585220	55588220	30.81	*EPS8L1*	EPS8-like 1
chr20	30223690	30226690	-26.72	*COX4I2*	Cytochrome c oxidase subunit IV isoform 2
chr20	45279100	45282100	25.21	*SLC13A3*	Solute carrier family 13
chr21	46338949	46341949	55.20	*ITGB2*	Integrin, beta 2

*Meth (T21 –N) represents the percent methylation difference between T21 twin and normal twin. The positive sign means T21 twin is more methylated than the Normal twin, and the negative sign means Normal twin is more methylated than T21 twin.

**Table 3:** GO terms associated with DMRs identified in MZ twins discordant for T21.

Biological Process	Gene Name	Gene Description	*P*	*FDR*
Embryonic organ morphogenesis	*HOXD3*	Homeobox D3	6.52E-06	1.60E-03
	*CTHRC1*	Collagen triple helix repeat containing 1		
	*USH1C*	Usher syndrome1C		
	*HOXB5*	Homeobox B5		
	*HOXB6*	Homeobox B6		
	*HOXD10*	Homeobox D10		
Embryonic skeletal system morphogenesis	*HOXD3*	Homeobox D3	2.79E-05	3.40E-03
	*HOXB5*	Homeobox B5		
	*HOXB6*	Homeobox B6		
	*HOXD10*	Homeobox D10		
Embryonic morphogenesis	*HOXD12*	Homeobox D12	7.85E-05	3.40E-03
	*HOXD3*	Homeobox D3		
	*HOXB5*	Homeobox B5		
	*HOXB6*	Homeobox B6		
	*HOXD10*	Homeobox D10		
	*CTHRC1*	Collagen triple helix repeat containing 1		
	*USH1C*	Usher syndrome1C		

*P*, nominal p-value.

*FDR*, False Discovery Rate- Benjamini–Hochberg.

**Table 4:** DNA methylation differences and gene expression fold changes in fibroblasts and iPSC of MZ twins discordant for T21; the genes listed are from Table 3.

	MZ twins discordant for T21 fibroblast cells		MZ twins discordant for T21 iPS cells	
Gene name	Methylation difference* (T21 twin-Normal twin)	Gene expression difference Log_2_ (T21/Normal)	Methylation difference* (T21 iPS–Normal iPS)	Gene expression difference Log_2_ (T21 iPS /Normal iPS)
*HOXD3*	35.2	-0.80	36.4	-5.7
*HOXD10*	62.16	-2.84	-6.8	-1.02
*HOXB5*	45.3	-0.32	59	-7
*HOXB6*	45.3	-1.85	59	-5
*CTHRC1*	31.3	-0.30	-53.4	0.43
*USH1C*	41.5	00.0	-5	0.4
*HOXD12*	60.58	3.12	77.92	-0.03
*Correlation*	-0.86		-0.56	

* Methylation difference is the percent methylation differences between fibroblast cells of T21 twin and Normal twin (column 2) and between iPS cells of T21 twin and Normal twin (column 4).

*Correlation* is the Pearson correlation between DNA methylation differences in column 2 and Log_2_ ratio of gene expression differences in column 3, and between DNA methylation differences in column 4 and Log_2_ ratio of gene expression differences in column 5.

**Table 5:** Histone modification and TFBS enrichment analyses of DMRs associated with T21.

**ENCODE Histone Modification gene-set library (Human)**			
**Histone Mark**	***P***	***FDR***	**Genes**
H3K27me3	9E-05	0.01	*CHST6;ESRP2;LAMC3;USH1C;EPS8L1;HOXD3;HOXD10;RNF130;ZNF212;PIK3R5*
H3K4me1	5E-04	0.03	*ISLR;CHST6;HSPB6;LAMC3;PHYHD1;ITGB2;PCDHGA1;HOXD10;ZNF212*
H2AFZ	2E-03	0.06	*DENND2C;PPP5C;ISLR;PHYHD1;RPL11;PCDHGA1;BCAS2;ZNF212*
**ENCODE TF ChIP-seq gene-set library (Human)**			
**TF**	***P***	***FDR***	**Genes**
CTCF	2E-04	0.1	*PPP5C;RPL11;ASB6;HOXD12;HDDC3;ZNF212;HOXB5;CTHRC1*
EZH2	1E-03	0.1	*PPP5C;CHST6;ESRP2;COX4I2;OTOF;EPS8L1;LOXL4;HDDC3;PIK3R5*

*P*, nominal p-value.

*FDR*, False Discovery Rate- Benjamini–Hochberg.

**Fig 5:**
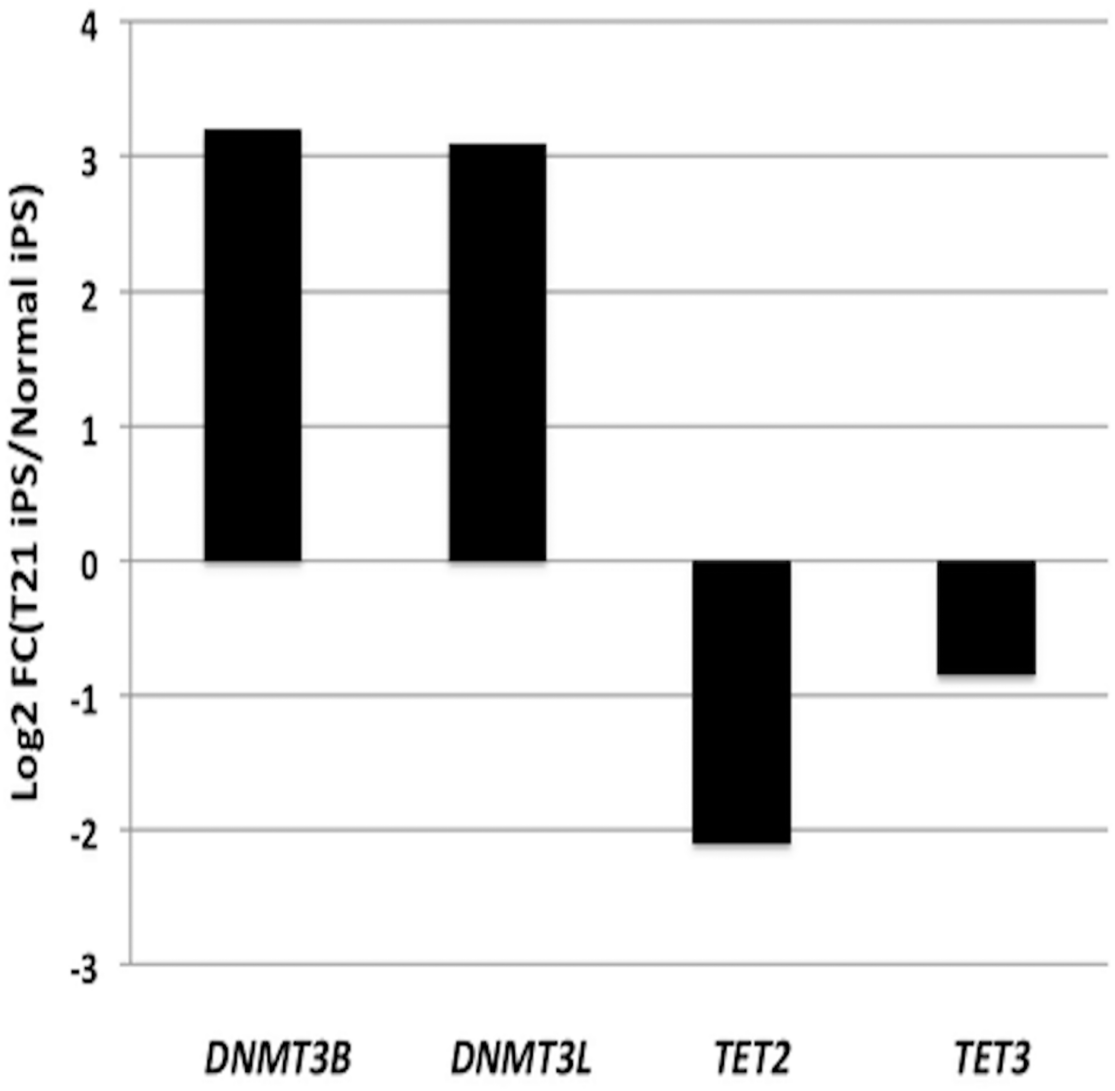
Gene expression fold changes of enzymes involved in DNA methylation. The log2 ratio of fold change is based on the average RPKM values of four normal twin iPS cell replicates and the average RPKM values of three T21 twin iPS cell replicates.

Furthermore, since the majority of the DMRs detected in the MZ twin discordant for T21 are hyper-methylated in T21, we subsequently evaluated the genes that are known to be directly involved in DNA methylation and de-methylation processes. The addition of methyl group to CpGs is controlled at several different levels and is carried out by a family of enzymes called DNA methyltransferases (DNMTs) (DNMT2, DNMT3L, DNMT3a and DNMT3b) [32]. On the contrary, active DNA demethylation is carried out by ten-eleven translocation (TET) enzyme-mediated oxidation (TET1, TET2 and TET3) [33]. Based on RNAseq data available from the iPS cells generated from the MZ twins discordant for T21 (55), we have observed an over-expression of DNA methyltransferase enzymes (DNMT3B and DNMT3L) and down regulation of TET2 and TET3 involved in DNA demethylation processes in T21 iPS cells compared to the normal twin iPS cells (Fig 5). It is of note that *DNMT3L* maps on chromosome 21, and its expression level in the T21 iPS cells is three fold greater than in the normal iPS cells. However, we did not observe expression changes of these genes in the skin fibroblast cell cultures. This may suggest that these epigenetic changes potentially happen in early embryonic stages [34].

### Methylation differences in T21 MZ twins discordant for CHD

We had also the opportunity to study two sets of T21 MZ twins discordant for CHD, and thus investigated DMRs that could potentially be linked to this common but heterogeneous congenital phenotype in DS. The first twin set was discordant for VSD (Samples 5 and 6) (Table 1), and the second set for AVSD (Samples 7 and 8) (Table 1). AVSD is the most common (43% of CHD cases in DS), and VSD is the second common cardiac defect in DS (32% of CHD cases in DS) [35]. Although genomic variation on chromosome 21 has been shown to modify the risk of CHD [3], in the case of MZ twins the genome is similar. Therefore, CHD may be the result of epigenetic alterations in concert with T21, or a stochastic event. We have identified 197 DMRs (25% methylation differences and q-value less than 0.001) in MZ twins discordant for VSD (Samples 5 and 6), and 88 DMRs in MZ twins discordant for AVSD (Samples 7 and 8), including 13 common DMRs between two sets. Table 6 shows the common DMRs observed in both twin sets and their association with different heart phenotypes based on previous studies [36–43]. However, the direction of DNA methylation is different for some of the gene promoters (*CBFA2T3*, *EPHA8*, *LY9*, *SLC9A3R2*) between VSD and AVSD (Table 6). This may suggest that the up or down regulation of these genes can lead to different heart phenotypes. The GO term analyses performed on genes harboring DMRs in MZ-twins discordant for AVSD (Samples 7 and 8), show an enrichment for endocardial cushion morphogenesis genes (adjusted *P* value 0.0091) and genes involved in metanephric nephron tube formation (adjusted *P* value 0.008) (Table 7). Additionally, we observed enrichment for genes involved in pyridine-containing compound metabolic process (adjusted *P* value 0.033) in MZ twins discordant for VSD (Samples 5 and 6) (Table 7). Furthermore, the top scores for TFBS enrichment analyses for DMRs in T21 twins discordant for AVSD highlighted EP300, E2F6, SMC3 and CEBPB binding sites, and for T21 twins discordant for VSD highlighted POLR2A (polymerase (RNA) II polypeptide A), CTCF and EP300 (Tables 8 and 9). Moreover, the top scores for histone modification marks enrichment analyses confirms the enrichment of marks associated with active enhancers such as H3K4me1 and H3K27ac in DMRs associated with both AVSD and VSD (Tables 8 and 9).

**Table 6:** Common DMRs observed in the two sets of T21 MZ twins discordant for AVSD and VSD.

Gene	Gene description	AVSD Twins	VSD Twins	Log_2_FC	Ref.
		Meth. differences	Meth. differences		
*PCDHGB1*	Protocadherin gamma subfamily B, 1	-44.44	-43.88	-1.59	-
*PFKFB2*	6-phosphofructo-2-kinase	29.67	92.29	-1.6	-
*TRPC3*	Transient receptor potential cationchannel	-26.14	-43.45	-0.86	Ref. (26,33)
*WDR27*	WD repeat domain 27	-29.23	-27.91	-1	-
*YOD1*	YOD1 deubiquitinase	29.66	92.28	-1.4	Ref. (28)
*CBFA2T3*	Core-binding factor, runt domain	26.46	-26.06	0.09	Ref. (27)
*CSRNP2*	Cysteine-serine-rich nuclear protein 2	26.21	55.58	-1.5	-
*DIP2C*	DIP2 disco-interacting protein 2 homolog C	-34.88	-25.81	-1.32	-
*EPHA8*	EPH receptor A8	30.86	-33.74	1	-
*LY9*	Lymphocyte antigen 9	-55.7	34.54	NA	-
*NFATC1*	Nuclear factor of activated T-cells	-30.08	-25.81	-1	Ref. (29,30)
*SLC9A3R2*	Solute carrier family 9	-38.78	32.26	-1.8	Ref. (31)
*TNNT3*	Troponin T type 3	-36.11	-59.85	0	Ref. (32)

Methylation differences show the DNA methylation percent difference between normal twin and twin with cardiac defect. Log_2_FC shows log_2_ fold change of RPKM value of gene expression in T21 normal twin over T21 twin with VSD. Gene expression data for T21 twins discordant for AVSD is not available. NA, Not available.

**Table 7:** GO terms associated with DMRs identified in T21 MZ twins discordant for CHD.

**T21 MZ twins discordant for AVSD**				
**Biological process**	**Gene**	**Gene description**	***P***	***FDR***
Endocardial cushion morphogenesis	*ENG*	Endoglin	4.08E-05	9.00E-03
	*TBX2*	T-box 2		
	*RBPJ*	Recombination signal binding protein		
	*SOX9*	SRY box 9		
Metanephric nephron tubule formation	*PAX2*	Paired box 2	8.01E-06	8.00E-03
	*SOX9*	SRY box 9		
	*PAX8*	Paired box 8		
**T21 MZ twins discordant for AVSD**				
Pyridine-containing compound metabolic process	*PNPO*	Pyridoxamine 5'-phosphate oxidase	4.64E-05	3.30E-02
	*GCK*	Glucokinase		
	*NUDT1*	Nudix		
	*NMRK2*	Nicotinamide riboside kinase 2		
	*NADK*	NAD kinase		
	*LDHB*	Lactate dehydrogenase B		

*P*, nominal *p*-value.

*FDR-BH*, False Discovery Rate- Benjamini–Hochberg.

**Table 8:** Histone modification and TFBS enrichment analyses of DMRs associated with VSD.

**ENCODE Histone modification gene-set library (Human)**			
**Histone Mark**	***P***	***FDR***	**Genes**
H2AFZ	3.2E-08	1.3E-05	*PXT1;AGPAT6;HDAC5;ITGB5;CCDC149;VPS4A;NAT10;NOD1;ASH2L;CKMT2;*
			*SEC14L1;FLAD1;WDR93;NDUFV3;IP6K1;ZNF687;ZBED3;CDKL3;TRPC3;*
			*SETDB1;PCDHGA1;YOD1;ISG15;CORO2A;ESPL1;ZSCAN23;L1TD1;CDK12;*
			*NIT1;SEC24D;HPD;LINC00346*
H3K4me1	3.0E-05	3.1E-03	*IFITM3;PXT1;CCDC149;SLC2A5;AQP3;PPCDC;CKMT2;KCTD20;C14ORF2;*
			*PPP6R2;KIAA0226L;CD14;SMYD3;NAE1;DIP2B;ZNF589;CDKL3;ACBD4;*
			*LYSMD1;DGKZ;GCFC2;SLC6A9;LUC7L3;STEAP1;HPD;CAPS2;*
H3K27ac	4.1E-05	3.4E-03	*CCDC101;PXT1;AGPAT6;NDUFA10;ISG20L2;ASH2L;C1ORF174;PHKG1;KCTD20;*
			*STK36;DGCR8;NDUFV3;ZNF687;ZBED3;GUSBP1;C8ORF59;SETDB1;ATAD5;*
			*LRBA;IFNGR2;LYSMD1;ISG15;SMU1;NBR2;NIF3L1;CFDP1;NHP2L1;ESPL1;*
			*NCL;MITD1;NAA16;ZNF414;CDK12;NIT1;STEAP1;C22ORF15;CAPS2;LINC00346*
**ENCODE TF ChIP-seq gene-set library (Human)**			
**TF**	***P***	***FDR***	**Genes**
POLR2A	7.7E-08	3.1E-05	*CSRNP2;FAF1;C15ORF61;ASH2L;ARID4B;C1ORF174;AQP3;PPCDC;UBL5;ALDH2;*
			*C14ORF2;FUNDC1;PPP6R2;DGCR8;ZNF687;NUDT12;TMPO;WDR38;CDKL3;*
			*GUSBP1;C8ORF59;TBCCD1;IFNGR2;TET2;YOD1;NIF3L1;NHP2L1;ESPL1;THRAP3;*
			*NCL;LUC7L3;ZSCAN23;HPRT1;STEAP1;HPD;PXT1;AGPAT6;TSSC1;VPS4A;NOD1;*
			*C17ORF51;OAZ2;SEC14L1;KCTD20;STK36;PNPO;SMYD3;NAE1;NDUFV3;*
			*ZNF589;IP6K1;NADK;TBC1D16;GINS1;ACBD4;CCDC12;ATAD5;LRBA;SGTA;*
			*LYSMD1;ISG15;SMU1;NUDT9;NBR2;STK24;CAPZA2;GNB4;MITD1;NAA16;*
			*ISG15;SMU1;NUDT9;NBR2;STK24;CAPZA2;GNB4;MITD1;NAA16;SEC24D;*
			*FERMT3;LINC00346*
EP300	1.1E-06	9.3E-05	*CSRNP2;FAF1;NDUFA10;C15ORF61;RPL10A;UBL5;ALDH2;FLAD1;FUNDC1;*
			*PPP6R2;ZNF687;TMPO;CDKL3;GUSBP1;IQCD;GCFC2;NIF3L1;CFDP1;FAM161A;*
			*NHP2L1;ESPL1;THRAP3;NCL;LGALS12;TLCD1;STEAP1;HPD;CCDC101;PXT1;*
			*TSSC1;NOD1;MTMR6;SOCS2;SEC14L1;KCTD20;STK36;SMYD3;NDUFV3;*
			*ZNF589;IP6K1;NADK;GINS1;SETDB1;CCDC12;ATAD5;LRBA;LYSMD1;SMU1;*
			*NBR2;TCEA2;MITD1;ZNF414;HIST1H4D;LINC00346*
CTCF	1.3E-06	9.4E-05	*BCAR3;CCDC101;AGPAT6;TSSC1;CCDC149;FAF1;VPS4A;NAT10;NOD1;*
			*RPL10A;AQP3;CX3CL1;MTMR6;SOCS2;KCNT1;SEC14L1;ALDH2;PHKG1;*
			*C14ORF2;FUNDC1;SMYD3;NAE1;DIP2B;ZNF589;WDR38;GUSBP1;ACBD4;*
			*SETDB1;SGTA;PCDHGA1;CORO2A;NUDT9;NBR2;ESPL1;CAPZA2;TLCD1;*
			*STEAP1;HPD;HIST1H4D;LINC00346*

*P*, nominal p-value.

*FDR*, False Discovery Rate- Benjamini–Hochberg.

**Table 9:** Histone modification and TFBS enrichment analyses of DMRs associated with AVSD.

**ENCODE Histone modification gene-set library (Human)**			
**Histone Mark**	***P***	***FDR***	**Genes**
H3K4me1	1E-05	0.006	*GABRB3;FAM49A;PLA2G12B;TPM3;KRT8;TMC6;TMPRSS13;ROCK1P1;*
			*RND1;HAUS1;LSM12;SPATA17;CCDC80;SUMO2;SLC26A9;MIR190B;ENG*
H3K27me3	6E-04	0.052	*GABRB3;FAM49A;PLA2G12B;TPM3;KRT8;TMC6;TMPRSS13;ROCK1P1;*
			*C9ORF129;F8;DLG3;PTCHD3;MIR190B;BCAS1;CES1*
**ENCODE TF ChIP-seq gene-set library (Human)**			
EP300	2E-05	0.018	*H2AFB1;CSRNP2;LUZP1;CSTF3;ATP5A1;GPATCH2;RPL36A;RND1;PIM2;*
			*ZNF585B;SLC35A2;MED1;TTC37;PTGIR;TIPIN;F8A1;HIST1H4K;KRT8;*
			*PDE4DIP;YOD1;MARCH8;TMC6;HAUS1;LENG1;LATS1;PAN2;CDC20B;PKIB;*
			*SPATA17;GSTA4;EBPL*
E2F6	2E-04	0.049	*TTC37;TIPIN;CSRNP2;ATP5A1;MARCH8;CLK2;HAUS1;LENG1;LATS1;PAN2;*
			*CDC20B*
SMC3	1E-04	0.049	*SLC35A2;TTC37;CSRNP2;RPL36A;KRT8;MIR449C;RND1;PKIB;SPATA17;*
			*GSTA4;ARSK; SERINC1;PIM2;LRRC4C;ZNF585B;BCAS1;CES1*
CEBPB	2E-04	0.049	*MED1;CSRNP2;LUZP1;RPL36A;YOD1;MAGT1;CMAHP;RND1;LENG1; LATS1;*
			*CDC20B;F8;LSM12;GSTA4;ARSK;SUMO2;EBPL;PIM2;ZNF585B;CES1*

*P*, nominal p-value.

*FDR*, False Discovery Rate- Benjamini–Hochberg.

## Discussion

DNA methylation analysis of fibroblasts from a rare pair of MZ twin discordant for T21, combined with normal MZ twins and T21 MZ twins as controls, revealed differences in promoter regions of genes involved in embryonic organ morphogenesis such as *HOXB5*, *HOXB6*, *HOXD3*, *HOXD10* and *HOXD12* in T21. Although the majority of these loci are hyper-methylated in the T21 compared to the normal twin, there is no enrichment for chromosome 21 genes. This highlights the point that the effect of the extra copy of chromosome 21 is not restricted to genes located on chromosome 21, and can modify the epigenetic status of loci located in the rest of the genome [2]. The fact that these gene regions are also differentially methylated in iPS cells suggests that these epigenetic marks are not tissue specific and are stably maintained.

*HOX* genes, transcription factors of the homeobox superfamily, are major regulators of animal development and regulate many biological processes including embryonic morphogenesis and differentiation [44,45]. Aberrant expression of *HOX* genes is associated with several abnormalities. For instance, altered expression of *Hoxb6* in mice has been associated with craniofacial abnormalities [46]. *HOXB5* encodes a protein that functions as a sequence-specific transcription factor that is mainly involved in lung and gut development [47]. Altered expression of *HOXB5* is linked to AML, bronchopulmonary sequestration and congenital cystic adenomatoid malformation tissue [48,49]. Also, the *HOXD* gene cluster has been associated with severe genital and limb abnormalities [50–52]. Moreover, in accordance with the observation that *HOXB* and *HOXD* gene clusters are down-regulated in T21, Billingsley C et al reported this observation when comparing 13 Ts65Dn (DS mouse model) and 11 euploid mouse embryos at 13.5 days gestation (E13.5) [53]. Altogether, these results indicate that dysregulation of *HOX* genes mediated by DNA methylation modification may play an important role in DS associated phenotypes.

Another observation in the DNA methylation analyses of MZ twins discordant for T21 is the presence of a higher level of DNA methylation in the genome of the T21 twin compared to the normal twin, both in iPSC and fibroblasts. This observation was also reported in unrelated T21 and normal individuals [12]. When the genome was dissected in different genomic features, the DNA hyper-methylation was even more pronounced in promoters and CpG islands. Therefore, it suggests that the presence of the extra copy of chromosome 21 in DS cases is associated with an increase in the overall genome methylation in T21. The hyper-methylation state of the T21 methylome versus the normal methylome in iPSC is interestingly associated with the up-regulation of DNA methyl-transferase genes (*DNMT3B* and *DNMT3L*), and down-regulation of DNA de-methylation genes (*TET2* and *TET3*) in T21 twin. However, it is still unclear if the presence of the extra copy of chromosome 21 *per se*, or specific gene(s) located on chromosome 21 is (are) leading this effect. It is of note that *DNMT3L*, a DNA methyltransferase regulator, maps on chromosome 21. Since DNA methylation patterns are established during early embryonic development [34], any changes in the methyltransferases activity can lead to disturbed methylation patterns in the cells [54]. DNMT3L increases the activity of DNMT3a and DNMT3b [54] that are the main enzymes required for *De Novo* DNA methylation and are extremely similar in structure and function [55,56]. However, it seems that DNMT3b is needed during early development, while DNMT3a is required for normal cellular differentiation [57]. Moreover, Baubec *et al* recently has shown the selective binding of DNMT3B to the bodies of transcribed genes, while excluded from active promoters and enhancers [58]. In accordance with the 3.1 fold change increase in the expression level of DNMT3L in T21 twin iPSC, we observed 3.2 fold change increase also in the expression level of DNMT3b in T21 iPSC compared to normal twin iPSC (Fig 5). These results suggest that the overexpression of specific genes on chromosome 21 (i.e., DNMT3L) (rather than the extra-material *per se*) leads to the hypermethylation of the genome in T21. However, further experiments are required to explore the association of DNMT3L and DNA methylation in the context of T21.

Moreover, we have observed enrichment for histone marks H3K27me3 and H3K4me1, and for CTCF and EZH2 binding sites in DMRs related to DS (Table 5). CTCF is a master regulator that plays a central role in multiple complex genomic processes, including transcription, imprinting, and chromatin interactions [59]. Recently, Wang et al reported a strong link between DNA methylation and the global occupancy patterns of CTCF. They have shown that the majority of CTCF binding sites are un-methylated and confirming an inverse relationship between methylation and CTCF occupancy [59]. EZH2 is also involved in maintaining the transcriptional repressive state of genes via H3K27me3 (repressive mark associated with constitutive heterochromatin), leading to transcriptional repression of the affected target gene. EZH2 also serves as a recruiting platform for DNA methyltransferase enzymes, thereby linking two epigenetic repression systems [60]. The fact that there is an enrichment for binding sites of CTCF and EZH2 which overlaps with the location of repressive histone marks H3K27me3 and H3K4me1 in DMRs associated with MZ twins discordant for T21, suggests that the altered methylation level in DNA sequence can potentially reflect the altered epigenetic architecture of chromatin structure. Whether DNA methylation alteration initiate the cascade of changes in the epigenetic architecture, or it is a result of changes in other epigenetic factors is not well known.

Furthermore, CHD is one of the most heterogeneous variable phenotypes in DS, and is present in 40% of the cases [35]. Recently, whole exome sequencing in a population of CHD cases pointed to the importance of epigenetic alterations in the pathogenicity of CHD in the general population [61]. Moreover, Serra-Juhé *et al* 2015 investigated the global methylation profile of fetal heart DNA in comparison to blood DNA from control subjects [62]. This study revealed a significant enrichment of differential methylation at genes related to muscle contraction and cardiomyopathies in the developing heart DNA in both isolated and syndromic heart malformations [62]. Since two sets of MZ twins in this study are concordant for T21, but discordant for CHD, they provide a unique opportunity to investigate the association of DNA methylation with the development of CHD in the context of T21. Interestingly, T21 MZ twins discordant for AVSD show enrichment for endocardial cushion morphogenesis genes (adjusted *P*-value 0.0091). This GO term includes *ENG*, *TBX2*, *RBPJ*, and *SOX9* (Table 7) that all are involved in heart development [63–65]. The MZ twins discordant for VSD show enrichment for the genes involved in pyridine-containing compound metabolic process (adjusted *P*-value 0.033) (Table 7). However, it is not known whether the pyridine-containing compounds are involved in heart development. Additionally, the ENCODE transcription factor binding enrichment analyses show a significant enrichment for binding sites of EP300 and its paralog CREBBP that are ubiquitously expressed transcriptional co-activators and histone acetyl transferases. Both of these factors are essential for normal cardiac and neural development [66]. On the contrary, E2F6 and CTCF binding sites that are also significantly enriched in DMRs associated with T21 twins discordant for AVSD, are transcription repressors [67]. E2F6 interacts with Dnmt3b and is required for binding of DNMT3B at CpG-islands [68], however it is not known that this interaction leads to the establishment or to the maintenance of DNA methylation.

In conclusion, the study of DNA methylation differences in MZ twins discordant for T21 revealed epigenetic alterations of gene promoters that are involved in embryonic organ morphogenesis and might be relevant to DS phenotypes. These DMRs are also targets of a common set of transcription factors and epigenetic regulators. In addition, we have observed an overall hypermethylation of DS genome compared to euploid normal genome, particularly in promoters and CpG islands. Moreover, the study of DNA methylation differences in T21 twins discordant for CHD highlighted epigenetic alterations of gene promoters that might be linked to the heart development. Furthermore, there is a significant enrichment for binding sites of EP300 and CREBBP that are important for heart development in both twins discordant for AVSD, and discordant for VSD. Altogether, the results of this study highlight the epigenetic effects of the extra chromosome 21 in T21 on loci outside of this chromosome that are relevant to DS associated phenotypes.

## Supporting Information

S1 Fig Correlation heatmap of two technical replicates for the MZ twins discordant for T21 methylome (Samples 1, 2, 3 and 4). For the normal twin (Samples 1 and 3) the correlation is 0.90, and for the T21 twin (Samples 2 and 4) the correlation is 0.91. The colors represent the local densities at each point in the scatterplot. The red line is linear regression fit and the blue line is loess fit.
(TIFF)

S1 Table Samples used for DNA methylation analyses and their sequencing information. Total reads, show the raw number of sequenced reads in million (M) obtained for each sample. Mapped, shows the number of reads uniquely mapped against the human genome hg19 in million (M).
(DOCX)

S2 Table The common number of DMRs in each sample set pair-wise comparison. (DOCX)

S3 Table Overlapping DMRs of table 2 with the previous studies of DNA methylation in DS. *Fetal Liver Mononuclear Cells. DS, Down syndrome. N, normal. M, Million. K, Thousands.
(DOCX)
