# Supplementary figures and images for: DNA-Methylation Patterns in Trisomy 21 Using Cells from Monozygotic Twins
Source: PLoS One. 2015 Aug 28;10(8):e0135555. doi: 10.1371/journal.pone.0135555 (PMC4552626; doi:10.1371/journal.pone.0135555)

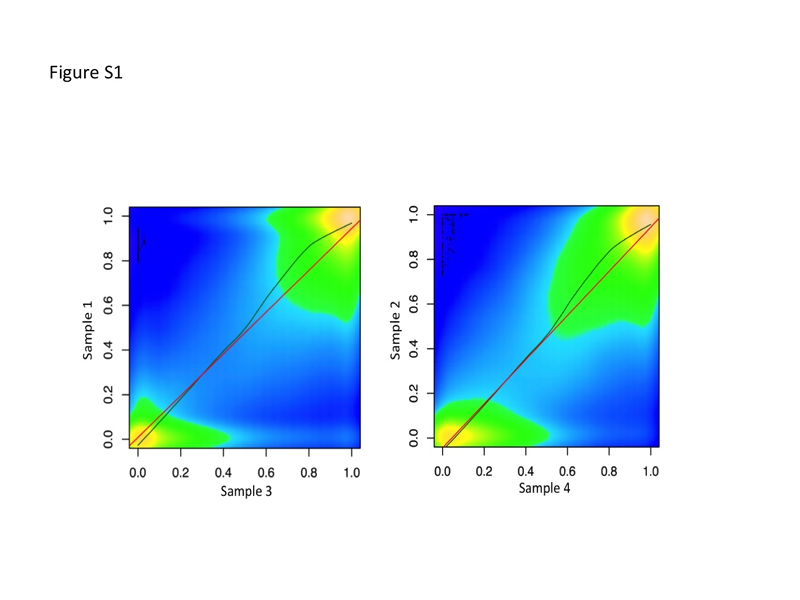

Supplement: S1 Fig — For the normal twin (Samples 1 and 3) the correlation is 0.90, and for the T21 twin (Samples 2 and 4) the correlation is 0.91. The colors represent the local densities at each point in the scatterplot. The red line is linear regression fit and the blue line is loess fit. (TIFF) [file pone.0135555.s001.tiff]
