# Supplementary material for: DNA-Methylation Patterns in Trisomy 21 Using Cells from Monozygotic Twins
Source: PLoS One. 2015 Aug 28;10(8):e0135555. doi: 10.1371/journal.pone.0135555 (PMC4552626; doi:10.1371/journal.pone.0135555)
Supplement: S1 Table — Total reads, show the raw number of sequenced reads in million (M) obtained for each sample. Mapped, shows the number of reads uniquely mapped against the human genome hg19 in million (M). (DOCX) [file pone.0135555.s002.docx]

| **Sample Set** | **Sample ID** | **Total reads** | **Mapped** | **Mapping efficiency** | **Number of CpG (20X)** |
| --- | --- | --- | --- | --- | --- |
| MZ twins discordant for T21 (Replicate 1) | Sample 1 | 104.3 M | 61.7 M | 0.61 | 2598760 |
|  | Sample 2 | 121.7 M | 71.7 M | 0.6 |  |
| MZ twins discordant for T21 (Replicate 2) | Sample 3 | 48.7 M | 25.1 M | 0.52 | 1167375 |
|  | Sample 4 | 47.4 M | 25.8 M | 0.55 |  |
| T21 MZ twins discordant for VSD | Sample 5 | 111.1 M | 63.5 M | 0.58 | 2187187 |
|  | Sample 6 | 107.5 M | 68 M | 0.64 |  |
| T21 MZ twins discordant for AVSD | Sample 7 | 149.9 M | 106 M | 0.71 | 4807733 |
|  | Sample 8 | 127 M | 94 M | 0.74 |  |
| Normal MZ twins pair 1 | Sample 9 | 130.1 M | 85.8 M | 0.65 | 3443437 |
|  | Sample 10 | 129.7 M | 84.8 M | 0.66 |  |
| Normal MZ twins pair 2 | Sample 11 | 163 M | 106.2 M | 0.65 | 4109993 |
|  | Sample 12 | 144.4 M | 95.0 M | 0.66 |  |
| Unrelated normal and T21 | Sample 13 | 108.9 M | 54.6 M | 0.51 | 2017353 |
|  | Sample 14 | 125.7 M | 72.6 M | 0.58 |  |
| iPS cells (MZ twins discordant for T21) | Sample 15 | 128 M | 71.2 M | 0.56 | 1481038 |
|  | Sample 16 | 141.9 M | 44.6 M | 0.33 |  |
|  | Sample 17 | 133.5 M | 73.8 M | 0.58 |  |
