# Supplementary material for: DNA-Methylation Patterns in Trisomy 21 Using Cells from Monozygotic Twins
Source: PLoS One. 2015 Aug 28;10(8):e0135555. doi: 10.1371/journal.pone.0135555 (PMC4552626; doi:10.1371/journal.pone.0135555)
Supplement: S2 Table — (DOCX) [file pone.0135555.s003.docx]

|  | (Samples 1,2,3,4)  DMRs=462 | (Samples 5,6)  DMRs=197 | (Samples 7,8)  DMRs=88 | (Samples 9,10)  DMRs=181 | (Samples 11,12)  DMRs=141 | (Samples 13,14)  DMRs=640 |
| --- | --- | --- | --- | --- | --- | --- |
| (Samples 1,2,3,4)  DMRs=462 | - | 43 | 07 | 15 | 16 | 74 |
| (Samples 5,6)  DMRs=197 | 43 | - | 13 | 08 | 21 | 88 |
| (Samples 7,8)  DMRs=88 | 07 | 13 | - | 08 | 11 | 07 |
| (Samples 9,10)  DMRs=181 | 15 | 08 | 08 | - | 58 | 27 |
| (Samples 11,12)  DMRs=141 | 16 | 21 | 11 | 58 | - | 44 |
| (Samples 13,14)  DMRs=640 | 74 | 88 | 07 | 27 | 44 | - |
