# Supplementary material for: DNA-Methylation Patterns in Trisomy 21 Using Cells from Monozygotic Twins
Source: PLoS One. 2015 Aug 28;10(8):e0135555. doi: 10.1371/journal.pone.0135555 (PMC4552626; doi:10.1371/journal.pone.0135555)
Supplement: S3 Table — *Fetal Liver Mononuclear Cells. DS, Down syndrome. N, normal. M, Million. K, Thousands. (DOCX) [file pone.0135555.s004.docx]

| **Study** | **Cells** | **Samples** | **CpGs** | **DMRs** | **Overlapping genes** |
| --- | --- | --- | --- | --- | --- |
| Jin *et al*. 2013 | Placenta | 11 DS vs 6 N | 1.5 M | 598 | *HOXD10, HOXD12* |
| Malinge *et al*. 2013 | FL-MNCs* | 7 DS vs 8 N | 200 K | 404 | *HOXB6* |
| Jones *et al*. 2013 | Buccal | 10 DS vs 10 N | 450 K | 3300 | *HOXB5, HOXB6* |
| Kerkel *et al*. 2010 | PBL | 29 DS vs 20 N | 27 K | 108 | *HOXB5* |
| Eckmann *et al*. 2012 | Placenta | 3 DS vs 47 N | 27 K | 304 | *HOXD10* |
